# Influence of strain on an ultrafast phase transition†

**DOI:** 10.1039/d2nr03395j

**Published:** 2022-12-05

**Authors:** Shaozheng Ji, Oscar Grånäs, Amit Kumar Prasad, Jonas Weissenrieder

**Affiliations:** Materials and Nano Physics, School of Engineering Sciences, KTH Royal Institute of Technology SE-100 44 Stockholm Sweden jonas@kth.se; Materials Theory, Department of Physics and Astronomy, Uppsala University Uppsala Sweden; Ultrafast Electron Microscopy Laboratory, The MOE Key Laboratory of Weak-Light Nonlinear Photonics, School of Physics, Nankai University Tianjin 300071 China

## Abstract

The flexibility of 2D materials combined with properties highly sensitive to strain makes strain engineering a promising avenue for manipulation of both structure and function. Here we investigate the influence of strain, associated with microstructural defects, on a photo-induced structural phase transition in Td–WTe_2_. Above threshold photoexcitation of uniform, non-strained, samples result in an orthorhombic Td to a metastable orthorhombic 1T* phase transition facilitated by shear displacements of the WTe_2_ layers along the *b* axis of the material. In samples prepared with wrinkle defects WTe_2_ continue its trajectory through a secondary transition that shears the unit cell along the *c* axis towards a metastable monoclinic 1T′ phase. The time scales and microstructural evolution associated with the transition and its subsequent recovery to the 1T* phase is followed in detail by a combination of ultrafast electron diffraction and microscopy. Our findings show how local strain fields can be employed for tailoring phase change dynamics in ultrafast optically driven processes with potential applications in phase change devices.

## Abstract 2

The flexibility of 2D materials combined with properties highly sensitive to strain makes strain engineering a promising avenue for manipulation of both structure and function.

## Graphical abstract

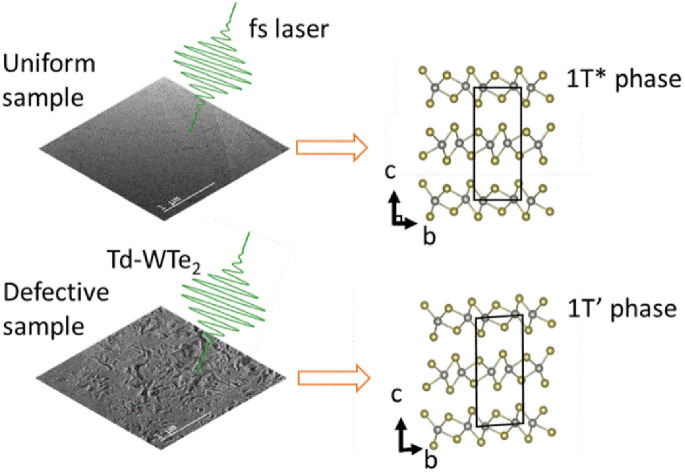

## 1. Introduction

Deliberate structural modulation of quantum materials using ultrafast perturbations is a highly coveted field in modern condensed matter physics, as it provides opportunities to uncover mechanisms behind photo-induced phase transitions (PIPT) and metastability of phases. It also opens opportunities in the design of next generation optical and quantum devices with improved functionalities. Transient excitation of materials through ultrashort laser pulses can result in the formation of exotic metastable phases,^1–9^ some with no direct analogue under equilibrium conditions. This can be employed to broaden the phase space of available material properties (*e.g.* electrical and optical), inspiring new applications in optoelectronics and quantum computing. One such example is the PIPT observed in the two-dimensional type-II Weyl semi-metal candidate Td–WTe_2_.^4,10^ Through a process including sliding of adjacent WTe_2_ layers the crystal structure transforms from a non-centrosymmetric (Td) phase towards to a metastable (1T*) phase with a partial recovery of inversion symmetry.^10^ The photo-induced metastable 1T* WTe_2_ phase can persist for nanoseconds before it relaxes back to its parent Td phase. The 1T* phase is distinct from the monoclinic 1T′ phase, in that it retains the orthorhombic structure of Td–WTe_2_. The formation of the 1T′ phase has not been observed in optically driven experiments, but can form under equilibrium conditions by application of static pressure^11,12^ and temperature.^13,14^ A recent analysis of the temperature induced Td to 1T′ phase transition in the iso-structural compound Td–MoTe_2_ revealed the existence of an intermediate orthorhombic phase.^15,16^ This intermediate phase has not been observed in studies of temperature driven Td to 1T′ transitions in WTe_2_,^13^ signifying the non-equilibrium character of the photo-induced 1T* phase. It is desirable to extend the properties available for deliberate optical switching to include transitions between all the phases (Td, 1T*, and 1T′) of WTe_2_. The three phases possess similar intra-layer atomic configurations but exhibit distinct layer stacking orders in the out of plane direction.^17^ The subtle adjustment in stacking order required for a change in symmetry is accompanied by dramatic modifications in properties, as evidenced by the changes in nonlinear optical properties,^4^ electronic band structure,^18^ ferroelectricity,^19,20^ topology^4,17,21^ and nonlinear Hall effect.^22–24^ PIPT can open an avenue for manipulation of these properties on an ultrafast timescale.^4^

Strain engineering of 2D materials is a promising venue for manipulation of atomic structure and properties.^25–30^ Ripple and wrinkle defects induce local strain fields in 2D materials.^31,32^ However, if local strain fields can be employed to manipulate PIPT processes in 2D materials, such as in Td–WTe_2_, requires experimental investigations. A study to address this open question should include real-space imaging at high spatial resolution to classify defect types and ultrafast temporal resolution to capture the evolution of the defects and the atomic structural dynamics necessary for a mechanistic description of the PIPT. The ultrafast electron microscope (UEM) is a tool satisfying such requirements. By using UEM, the ultrafast dynamics of acoustic phonon propagation in two-dimensional materials have been studied and demonstrate the influence of defects or local strain fields.^33–36^ Recently, coupling of photo-induced excitation of acoustic phonons and periodic lattice distortions (PLDs) has been reported in the charge density wave material 1T–TaS_2_.^37^ These seminal studies have demonstrated the capacity of UEM in determining the structural response and dynamics on relevant length and time scales. Here we take advantage of the high spatial and temporal resolutions of an ultrafast electron microscope, by combining ultrafast electron diffraction and imaging techniques, to study the role of defects and the associated localized strain on PIPT dynamics in Td–WTe_2_. We observed that in uniform (low-defect density) samples, the PIPT involves an orthorhombic Td to a metastable orthorhombic 1T* phase transition completed within 20 ps. The metastable orthorhombic 1T* phase is stabilized for at least 100 ps. Samples prepared with local strain fields, induced by wrinkle defects, exhibit a distinctly different phase transition process, where we observe formation of a metastable monoclinic 1T′ phase within 70 ps. The 1T′ phase is then partially recovered to 1T* in a process that is influenced by the evolution of the local microstructure in the sample.

## 2. Results and discussion

### Structure evolution revealed by ultrafast electron diffraction

**Fig. 1:**
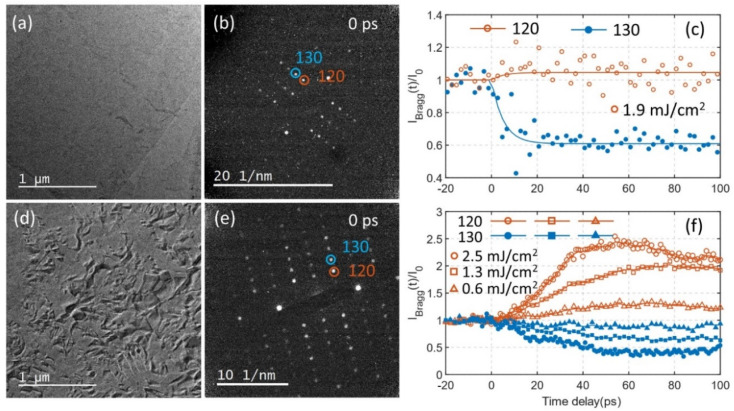
(a) TEM image of a uniform Td–WTe_2_ sample. (b) The corresponding electron diffraction pattern at the [001] zone axis (with 120 and 130 spots indicated by red and blue circles). (c) Temporal evolution of the 120 and 130 diffraction intensities following excitation by 1.9 mJ cm^−2^ laser fluence at 515 nm. (d) TEM image of a sample prepared with wrinkle defects (a high-resolution TEM image is presented in the ESI Fig. S1†). (e) Diffraction pattern from the area in (d). The 120 and 130 spots are encircled. (f) Temporal evolution of the 120 and 130 diffraction intensities for pump fluencies 0.6, 1.3, and 2.5 mJ cm^−2^.

We begin by comparing the photo-induced structural response in uniform (non-defective) samples with that of samples containing wrinkle defects. In our recent work, we found that optical excitation at 515 nm drives the orthorhombic Td–WTe_2_ ground state phase into a related orthorhombic phase, 1T*, with a ∼5 ps time constant.^10^Fig. 1 show a transmission electron microscopy (TEM) image (a), representative diffraction pattern (b), and temporal evolution of electron diffraction intensity (c) after photoexcitation of a uniform sample. The diffraction pattern is obtained at the [001] zone axis from a selected sample area with diameter of 1.2 μm and the sample was excited with a 1.9 mJ cm^−2^ laser fluence at 515 nm. The 120 and 130 diffraction spots show opposite temporal evolution in diffraction intensity (increase *vs.* decrease after time zero). The time constants extracted from a single exponential fitting were ∼5 ps for both spots. The increase in the 120 diffraction intensity is less than 10 percent, while the intensity of the 130 spot decrease by 40 percent. After 20 ps time delay the intensities become nearly constant and remains at the same level for at least 100 ps. The observed changes in intensity and dynamics is in agreement with a PIPT from Td to 1T* phase.^10^

A TEM image of a sample containing wrinkle defects are shown in Fig. 1d. The wrinkle defects introduce local bending and strain fields, which appears as orientational contrast in the image. A high-resolution TEM image of the wrinkle defect structure is shown in the ESI Fig. S1.† The image reveals a slight orientational difference between areas separated by a defect. The diffraction analysis for this sample was also conducted at the [001] zone axis (Fig. 1e), on average. Time-resolved diffraction intensities of the 120 and 130 spots are shown in Fig. 1f (at the pump fluences 0.6, 1.3, and 2.5 mJ cm^−2^). The temporal dynamics of spots 120 and 130 show striking difference in the sample containing wrinkle defects when compared to the uniform sample. The transient change in diffraction intensity persists over significantly longer time scales, *i.e.* slower processes not observed for the uniform sample are induced in the defective sample. Slow structural dynamics was observed at all three pump fluences. The diffraction intensity of the 120 spot increased significantly compare to the uniform sample (compare Fig. 1(c) and (f). At 2.5 mJ cm^−2^ pump fluence, for the sample containing wrinkle defects, three regions can be distinguished in the intensity trace of the 120 spot. In the first region, from time delay 0 ps to approximately 40 ps, the intensity increases almost linearly with time. At around 40 ps an inflection point is observed, after which the intensity increases at a slower rate. The intensity reaches a maximum at around 60 ps, after which the intensity slowly begins to decrease. The same trend can also be observed in the first order derivative of the 120 intensity as a function of time (Fig. S2†), where a steep decrease in rate is detected in the range 30 to 40 ps. The temporal intensity traces for the 120 spot at 0.6 and 1.3 mJ cm^−2^ laser fluence also exhibit three distinct regions. The inflection points at around 40 ps is not as distinct as in the 2.5 mJ cm^−2^ trace, mostly because of the lower rate at which the intensity change between 0 ps and 40 ps. Maximum intensity is reached between 60 ps and 80 ps, slightly later than that of 2.5 mJ cm^−2^ laser fluence. After reaching the maximum, the intensity slowly decreases with time. The intensity of the 130 spot exhibits almost opposite behavior to that of the 120 spot. Note that the all the symmetric 120 diffraction spots (120, 12̄0, 1̄2̄0 and 1̄20) exhibit similar evolution with time and this is also true for 130 and its symmetric spots (Fig. S3†). This combined behavior cannot be explained by a rigid orientational change of the sample, instead it should be interpreted as a change in crystalline structure. Temporal traces extended to 600 ps are shown in Fig. S4† for 1.3 mJ cm^−2^ and 0.6 mJ cm^−2^ pump fluences.

**Fig. 2:**
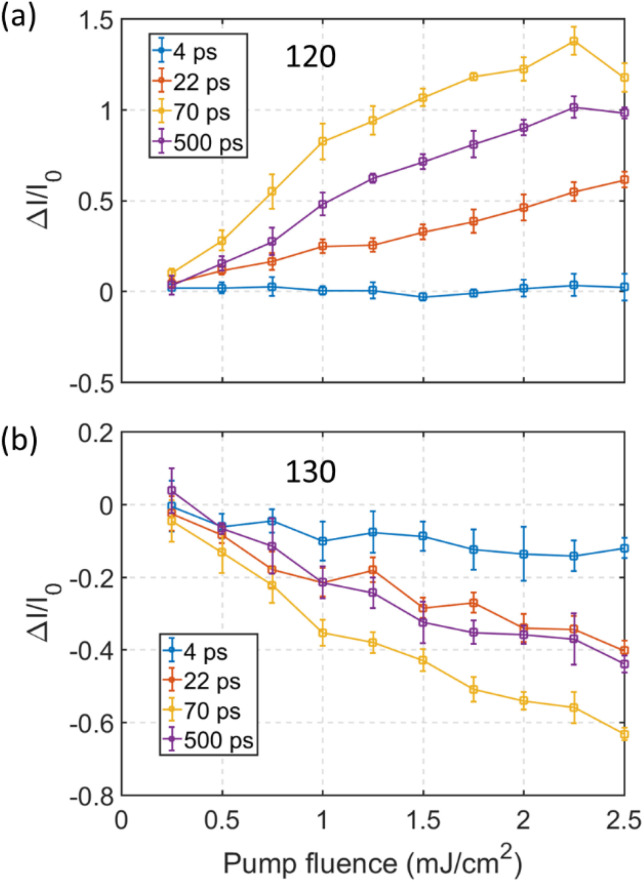
Pump fluence dependent changes in diffraction intensity of (a) the 120 and (b) the 130 diffraction spots for a sample prepared with wrinkle defects. The color codes represent change in intensity normalized to before time zero at 4 ps (blue), 22 ps (red), 70 ps (yellow), and 500 ps (violet) time delay. Data points are averaged from five experimental data sets and the error bars represent the standard deviation.

The relative change in intensity of the 120 and 130 spots increases with pump fluence. A detailed pump fluence dependence of the diffraction intensity for the 120 and 130 spots, at several time delays, is shown in Fig. 2. The behavior of the 140 spot resembles that of the 120 spot and the 150 spot acts similar to the 130 spot (shown in ESI Fig. S5†). At a pump fluence below 1 mJ cm^−2^ the intensity of the 120 spot at 22 ps, 70 ps and 500 ps increases almost linearly (Fig. 2(a)). Conversely, the intensity of the 130 spot decreases linearly with increasing pump fluence (Fig. 2(b)). At 22 ps delay the rate of increase in intensity is almost constant with fluence, tentatively a slight decrease in rate can be observed at around 1 mJ cm^−2^. Inflection points are observed for the 120 spot at around 1 mJ cm^−2^ in the traces for 70 ps and 500 ps temporal delay. Above the inflection point the rate of increase of the 120 spot intensity is reduced but still exhibits a linear trend with increasing pump fluence up to ∼2.2 mJ cm^−2^. Note that the gap between 22 ps and 70 ps increases linearly before 1 mJ cm^−2^, while no significant change in gap can be detected at pump fluences above 1 mJ cm^−2^. It is worth noting that 1 mJ cm^−2^ is the threshold pump fluence for the photo-induced phase transition from Td–1T* phase as reported in ref. 10. The decay in intensity of the 120 spot at long delays (after 70 ps in Fig. 1f) is reflected by the gap opening between the 70 ps and 500 ps curves in Fig. 2a. Interestingly, the gap between the 70 ps and 500 ps curves increase below a fluence of 1 mJ cm^−2^ but after remains almost constant. This is also true for the 130 spot. Although a difference is that the intensity for the 130 spot at 500 ps delay is nearly the same as what is observed at 22 ps delay while for the 120 spot the intensity at 500 ps is still large compared to that observed at 22 ps. Together, this indicates that the photoexcited structural relaxation process from 70 ps to 500 ps is similar at pump fluencies larger than 1 mJ cm^−2^.

**Fig. 3:**
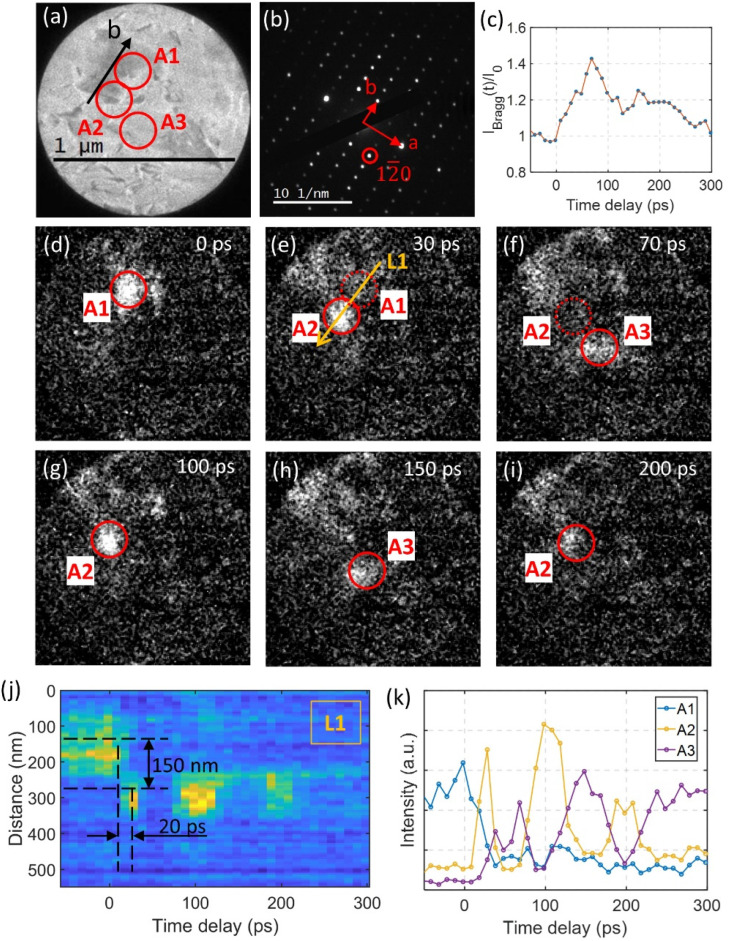
(a) Bright field image of a sample prepared with wrinkle defects. (b) Selected area diffraction pattern of the region shown in (a). (c) Temporal intensity evolution of the 12̄0 spot. (d)–(i) Show time resolved dark field images from the selected 12̄0 spot indicated in (b). (j) The time resolved intensity along the line L1 as marked in (e). (k) Time dependent integrated intensity from the selected regions labeled A1, A2, and A3 in (d) to (i). A pump laser fluence of 1.9 mJ cm^−2^ was used in the experiments.

The combination of the time-resolved intensity evolution of the diffraction spots shown in Fig. 1(f) and the pump fluence dependent results at selected time delays shown in Fig. 2 implies that the structural evolution following the photoexcitation involves at least three structural reorganization processes in samples prepared with wrinkle defects. This must be closely linked with the local defect structure since only the Td to 1T* transition is observed for uniform samples as shown in Fig. 1(c). By taking the advantage of microscopic capacity of UEM, we obtained time resolved dark field images to trace the spatial origin of the transient diffraction intensity in real space. The results are summarized in Fig. 3.

### Local microstructure evolution revealed by ultrafast dark-field imaging

The bright field image shown in Fig. 3(a) provides insights to the local microstructure of a region of interest in the defective sample. By selecting the 12̄0 spot encircled in the diffraction pattern shown in Fig. 3(b), dark field images recorded at different time delays following ultrashort laser excitation at pump fluence of 1.9 mJ cm^−2^ are shown in Fig. 3(d)–(i). A video compiled from the dark field results is included in the ESI (ESI 1†). At 0 ps delay, the main contribution to the intensity of the 12̄0 spot originates from the bright region labeled A1. The intensity contribution is mainly determined by the orientation of the local wrinkle defects. The wrinkle defects introduce local orientational deviations in the sample, as can be observed in the high-resolution TEM images in Fig. S1.† After photoexcitation, the intensity of the spot 12̄0 increases until approximately 70 ps (Fig. 3(c)). Note that the normalized intensity at its maximum (at 70 ps delay) only increases with approximately 40%, less than what is shown in Fig. 2(a). This discrepancy is a result of the lower wrinkle defect density within the observation region, as the change in normalized intensity of the diffraction spot is highly correlated with the wrinkle defect density. During the initial 70 ps time window we observe two processes in the dark field images. A snapshot from the first process is shown in Fig. 3(e), where the region labeled A2 becomes increasingly brighter simultaneous as the A1 region fades. The direction from A1 to A2 is aligned precisely along the *b* axis in the sample (L1). The second process proceeds from 30 ps to 70 ps. Now region A2 fades while the region labeled A3 becomes brighter (Fig. 3(f)). The temporal intensity evolutions of regions A1, A2, and A3 are shown in Fig. 3(k). The time step between data points is 10 ps and the intensity shown is integrated over 8 × 8 pixels (40 × 40 nm^2^ region). Note that the intensity of the A2 region do not increase immediately after time zero but with a time delay of ∼10 ps. The intensity of region A2 reaches its maximum at 30 ps which means that the rise time to maximum intensity is within a 20 ps time duration. By following the temporal intensity along the line L1 indicated in Fig. 3(e), we observe no significant intensity change within the first 10 ps after time zero at 300 nm longitudinal position which corresponds to the position of the A2 region on the line (see Fig. 3(j)).

The intensity change trace from the position at 150 nm (corresponding to region A1) to the position at 300 nm (A2) is weak. This may indicate that the intensity transfer from A1 to A2 is not an in-plane transportation but rather involve out-of-plane lattice deformations. After the intensity transfer to region A2 (at time delay >30 ps), the regions A2 and A3 show out-of-phase intensity oscillations with a period of approximately 100 ps (see Fig. 3(k). After two periods, the oscillation is damped out. This happened simultaneous to a decrease in the diffraction intensity of the 12̄0 spot as shown in Fig. 3(c). The oscillation in intensity between A2 and A3 can be explained by a mechanism where acoustic phonons are launched after an initial photoinduced change in structure (see *e.g.* ref. 38).

### Photo-induced driving force of local microstructure evolution

From the dark field analysis, we can correlate the complex diffraction intensity dynamics in Fig. 1(f) and 3(c) to the real space evolution in microstructure. We observe that the three-stage dynamics following photo-excitation is highly correlated with the defective microstructure and its evolution. As mentioned before, the diffraction results cannot be explained by a simple rigid orientational change of the sample, instead it should be interpreted as a change in structure (also further demonstrated by the simulations provided in Fig. S8†). It can be inferred that the microstructure evolution is coupled with the change in crystalline structure. In the following, we will discuss what is driving the evolution in microstructure.

As previously reported,^4,10,18^ optical excitation can launch a ‘shear’ phonon (A_1_ optical phonon at ∼0.23 THz) in WTe_2_. The atomic motions inflicted by the A_1_ optical phonon can be described as anti-phase motion of adjacent layer of WTe_2_ along the *b* axis. To determine if the A_1_ optical phonon is excited in connection with the Td to 1T* phase transition in a sample containing wrinkle defects, we re-examine the evolution in diffraction intensity of the 130 spot at 2.5 mJ cm^−2^ pump fluence in the range from −5 ps to 30 ps (Fig. S6†). An intensity decay corresponding to the Td to 1T* phase transition is observed, same as for the photo driven phase transition in an unstrained sample. This shows that the Td to 1T* phase transition also occurs in a strained sample and already at 15 ps the crystal structure has reached the coordinates of the 1T* phase. We observe a weak intensity oscillation in the first 6 ps and following even weaker oscillations indicates that the A_1_ optical phonon is strongly damped in strained samples. From our analysis of the time resolved dark field imaging results (Fig. 3(j) and (k)) we bring that during the first 10 ps after time zero there is no significant intensity transfer from region A1 to region A2. This implies that the microstructure has yet to be changed. During the following 10 ps we observe a reduction in the intensity contribution from region A1 (Fig. 3(e)) simultaneous to an intensity increase at region A2. Taken together, the results infer that the initial excitation of the A_1_ optical phonon and the Td to 1T* phase transition, with its intrinsic shear displacement of the layers along the *b* axis, modulates the local strain field. This modulation of the local strain field will then activate a structural change at the wrinkle defects. This interpretation is consistent with that the temporal microstructure evolution from region A1 to region A2 observed in the dark field analysis in Fig. 3(e) is exactly aligned along the *b* axis. We can therefore conclude that the evolution in microstructure is the result of strain field redistribution induced by the photoexcitation process. The result is complemented by our temperature dependent dark field imaging analysis of a sample prepared with wrinkle defects. Interestingly, we do not observe any change in microstructure in the temperature range between 298 to 398 K (Fig. S7†), showing that this process is only accessible through optical excitation.

### Formation of metastable 1T′ phase

**Fig. 4:**
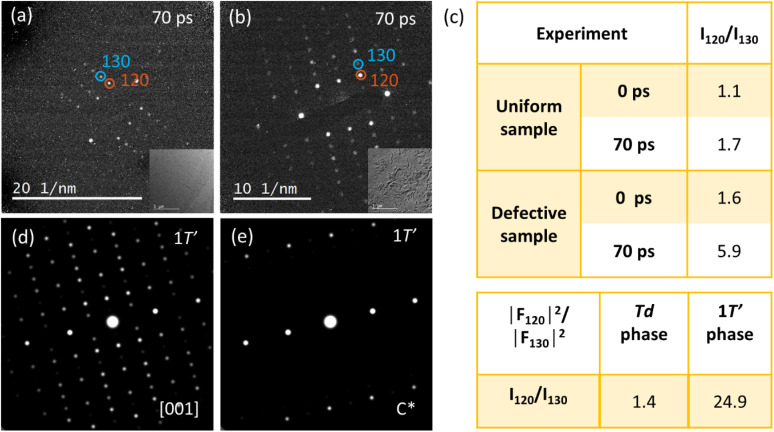
Diffraction patterns collected at 70 ps temporal delay from (a) a uniform sample and (b) a sample containing wrinkle defects. (c) List of the integrated intensities ratio of the 120 and 130 spots at 0 ps and 70 ps time delay from uniform sample and wrinkle sample. And the calculated intensity ratio *I*_120_/*I*_130_ based on structure factors from the Td and 1T′ phases. (d) Simulated diffraction pattern from the monoclinic 1T′ phase of WTe_2_ at the [001] zone axis of 1T′. (e) Simulated diffraction pattern aligned to the *c* direction of the Td/T* phases (*i.e.* reciprocal vector c* direction of 1T′).

As shown Fig. 1, the crystal structure of the wrinkle defective sample 70 ps after photoexcitation is different from the 1T* phase obtained by excitation of a uniform sample. Now we turn our attention to determining the photo-induced structural changes observed in samples with wrinkle defects. We analyzed diffraction patterns from uniform and wrinkle defective sample collected at 70 ps delay at a pump fluence of 1.9 mJ cm^−2^ (Fig. 4(a) and (b)). As described in conjunction with Fig. 2 and S5,† samples prepared with wrinkle defects exhibits a dramatic increase in intensity of the 120 and 140 spots at 70 ps, while the 130 and 150 spots show a significant decrease in intensity. In the 70 ps diffraction pattern from the defective sample (Fig. 4(b)) we observe that the 120 spot and its symmetric spots (12̄0, 1̄2̄0, and 1̄20) all become much brighter than the 130 spot and its symmetric spots, which rules out the possibility of sample tilting. To more clearly illustrate the difference in structure between the uniform samples and samples prepared with wrinkle defects we calculated the relative integrated intensities, *I*_120_/*I*_130_, at 0 ps and 70 ps (Fig. 4(c)). *I*_120_/*I*_130_ in the wrinkle defective sample is 1.6 at 0 ps time delay. This is significantly higher than what was found in the uniform sample (1.1) and is a direct result of local bending and strain (the presence of a wrinkled microstructure). At 70 ps time delay *I*_120_/*I*_130_ increases to 1.7 in the uniform sample. Such change in relative intensity can be rationalized as due to the expected optically driven structural phase transition from Td to 1T*. In the 1T* phase *I*_120_ is expected to increase while *I*_130_ should decrease in intensity.^10^ In the sample with wrinkle defects *I*_120_/*I*_130_ increases to an even higher value (5.9).

Inspired by the temperature induced Td to 1T′ phase transitions observed for Td–WTe_2_ ^13,14^ and Td–MoTe_2_,^39^ we investigate if the structure formed at 70 ps delay belongs to the 1T′ phase. Based on our first-principles calculations we can conclude that the 1T* and the 1T′ phases are close in energy. Depending on the treatment of dispersion forces (see computational methods), the energy difference between the two phases range between degenerate (∼1 meV per formula unit) to 4 meV per formula unit in favor of 1T*. This is in agreement with previous calculations with similar methodology by Sie *et al.*^4^ Energy barriers are unfortunately too computationally expensive to estimate for the resources available. The weak van der Waals bonding of the layers and that the transition can be thermally exited above approximately 565 K^13^ suggests that the barriers are not prohibitive. The diffraction pattern collected from a sample prepared with wrinkle defects at 70 ps delay (Fig. 4b) is similar to the monoclinic 1T′ phase of WTe_2_ at the [001] zone axis. As a comparison we show a simulated diffraction pattern of 1T′–WTe_2_ in Fig. 4d (coordinates from DFT). From the simulation we can immediately recognize strong 120 and 140 spots while the 130 and 150 spots are significantly weaker. In the analysis of the diffraction results it appears that the presence of wrinkle defects in Td–WTe_2_, and the local strain fields associated with such defects, generate favorable conditions for formation of a metastable monoclinic phase similar to 1T′–WTe_2_. However, the ratio *I*_120_/*I*_130_ at 70 ps (5.9) is not as high as what would be expected of a pure 1T′ phase (24.9). This can be explained by that a significant portion of the sample remains in the T* phase. That only a fraction of the sample transitions to the 1T′ phase can be rationalized with a mechanism where the transition is highly correlated with the local strain field present at the wrinkle defects, as is supported by the dark field image shown in Fig. 3. Note that the WTe_2_ diffraction pattern at 70 ps is similar to the monoclinic phase at the [001] zone axis, but not to the simulated pattern along the c* direction (out-plane primitive wavevector for the reciprocal lattice, 3.4° off [001], simulation shown in Fig. 4e).

**Fig. 5:**
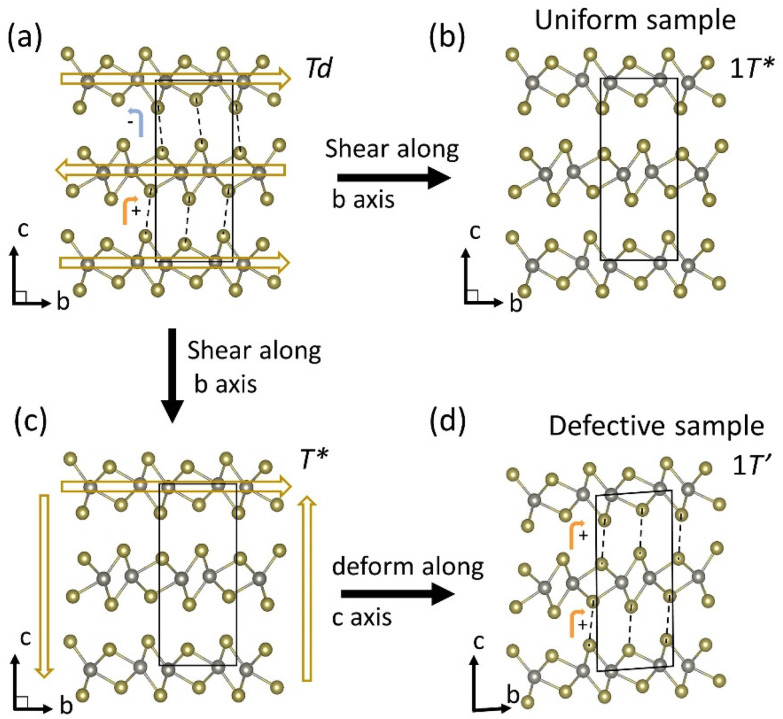
(a) to (b) illustrates the photo induced phase transition pathway from the non-centrosymmetric Td phase to the centrosymmetric 1T* phase in a uniform sample. (a)–(c)–(d) shows the pathway for the photo driven Td to 1T′ phase transition in samples prepared with wrinkle defects.

The analysis of the electron diffraction results from a sample containing wrinkle defects at 70 ps delay following photoexcitation indicates that the Td–WTe_2_ lattice may follow a path to a metastable monoclinic 1T′ phase. A tentative mechanism for the phase transition pathway is illustrated in Fig. 5(a)–(d). The material will first complete a Td to 1T* phase transition. This transition is completed within the first 15 ps following photoexcitation. Due to the presence of local wrinkle defects and associated pinning sites, the sample will experience a change in local strain field which results in a deformation of the sample along the *c* axis. The layers will simultaneously shear along the *b* axis to complete the phase transition to a metastable 1T′ phase. The new stacking orientation is illustrated in Fig. 5(d). Note that the dashed lines connecting protruding Te atoms in neighboring WTe_2_ layers are now oriented in the same direction in-between the top and middle layers as in-between the middle and bottom layers of the unit cell. Evidence for the deformation along the *c* axis can be found in the observed [001] zone axis diffraction pattern for the 1T′ phase at 70 ps delay as discussed in connection with Fig. 4. The orientation of sample will on average be similar to what is shown in Fig. 5(d), with the probing electron trajectory parallel to the *c* axis of the 1T′ phase.

## 3. Conclusions

The influence of local microstructure on the PIPT process in Td–WTe_2_ is determined by a combination of ultrafast electron diffraction and microscopy. Photoexcitation of uniform samples exclusively launch a Td to 1T* phase transition. Interestingly, samples prepared with wrinkle defects exhibit a different process that can be closely correlated to the local microstructure. The change in local strain field induced by the photo-induced launching of an A_1_ phonon combined with the shearing of adjacent layers by the Td to 1T* phase transition places wrinkle defective samples on a path towards formation of a metastable monoclinic 1T′ phase. The second phase transition is closely coupled with the evolution of the local microstructure and strain field. The conclusions of this work point to the feasibility of employing deliberate design of microstructures to control the PIPT process in Td–WTe_2_. Strain field modulation by ultrafast laser pulses may tentatively be employed as an efficient method for ultrafast manipulation of local material properties in 2D quantum materials.

## 4. Methods

### Experimental methods

Single crystal Td–WTe_2_ samples (2D Semiconductor, USA) were prepared for transmission electron microscopy (TEM) by careful slicing along the layers using an ultramicrotome (Leica) with a diamond knife. The samples were subsequently placed on single layer graphene TEM grids (Ted Pella). The area we selected for diffraction and imaging is on a micrometer scale. On this length scale, uniform sample regions (with no apparent defect contrast) and defected sample region can be selected. The TEM images show examples of images from low-defect-density and high-defect-density regions are shown in Fig. S9.† The sample thickness was estimated from electron energy-loss spectroscopy and is approximately 20 nm. Ultrafast time-resolved electron diffraction and microscopy analysis was conducted using an UEM.^40^ The UEM is based on a modified JEOL JEM 2100 TEM interfaced with a femtosecond laser source (1030 nm, 300 fs, Tangerine HP, Amplitude Systemes) and operates in a pump–probe mode at 200 keV. Synchronized second (515 nm) and fourth harmonic (258 nm) laser beams are used for photoexcitation and generation of photoelectron probe pulses. The sample was excited at a 70 kHz repetition rate with laser fluence of 0.6–2.5 mJ cm^−2^ with a laser focal spot of approximately 120 μm in FWHM. The temporal FWHM of the probe electron pulses was estimated to 1.4 ps by photo induced near field electron microscopy analysis on silver nanowires.^41^ A mechanical linear delay stage (Newport ESP301) was used to control the relative time delay between the arrival of the pump and probe pulses. The high-resolution TEM analysis was performed using a Thermo Fischer Themis Z operated at 300 kV. The microscope is equipped with a spherical aberration corrector (CEOS CETCOR), which compensates aberrations up to 3rd order. HRTEM images were acquired by a Gatan OneView camera. Temperature controlled electron diffraction and dark field analysis were carried out using a Gatan double tilt heating holder (model 652) from room temperature to ∼400 K.

### Computational methods

Density functional theory (DFT) was used to calculate the structure and energy of the 1T* and 1T′ structures of WTe_2_. Advised by previous work, indicating a strong dependence of the structure on the treatment of dispersion forces when performing structural relaxations,^4^ we bracket our result by using the generalized gradient expansion by Perdew, Burke and Ernzerhof^42^ together with (1) no additional treatment of dispersion forces, (2) dispersion forces according to Grimme D2,^43,44^ (3) dispersion forces according to Grimme D3,^45^ and (4) dispersion forces according to Grimme D3 including Becke Johnson damping.^46^ We use the projector augmented plane-wave formalism as implemented in the Vienna *ab initio* Simulation Package (VASP).^47,48^ We use a Γ-centered *k*-point grid of 18 × 10 × 5 *k*-points for the 1T′ as well as the 1T*. The energy and forces were sufficiently converged at a plane-wave cut-off of 278 eV. The forces are relaxed to 1 × 10^−6^ eV Å^−1^. The 1T′–WTe_2_ structure obtained from DFT was used as input for simulations of diffraction patterns by Crystal Maker SingleCrystal 3.

## Author contributions

Shaozheng Ji and Amit Kumar Prasad performed the ultrafast electron diffraction and ultrafast dark-field imaging experiments. Oscar Grånäs conducted the DFT simulations. Jonas Weissenrieder initiated the research and supervised the project. Shaozheng Ji wrote the manuscript with input from all authors.

## Conflicts of interest

There are no conflicts to declare.

## Supplementary Material

NR-015-D2NR03395J-s001

NR-015-D2NR03395J-s002
