# Supplementary material for: Influence of strain on an ultrafast phase transition
Source: Nanoscale. 2022 Dec 5;15(1):304–12. doi: 10.1039/d2nr03395j (PMC9773179; doi:10.1039/d2nr03395j)
Supplement: NR-015-D2NR03395J-s001 [file NR-015-D2NR03395J-s001.pdf]

Electronic Supplementary Material (ESI) for Nanoscale.
This journal is © The Royal Society of Chemistry 2022

Supporting Information to:

Influence of Strain on an Ultrafast Phase Transition

Shaozheng Ji[1,3], Oscar Grånäs[2], Amit Kumar Prasad[1] and Jonas Weissenrieder[1*]

[1]Materials and Nano Physics, School of Engineering Sciences, KTH Royal Institute of Technology, SE-100 44 Stockholm, Sweden

[2]Materials Theory, Department of Physics and Astronomy, Uppsala University, Uppsala, Sweden

[3]Ultrafast Electron Microscopy Laboratory, The MOE Key Laboratory of Weak-Light Nonlinear Photonics, School of Physics, Nankai University, Tianjin 300071, China

Supporting Figures

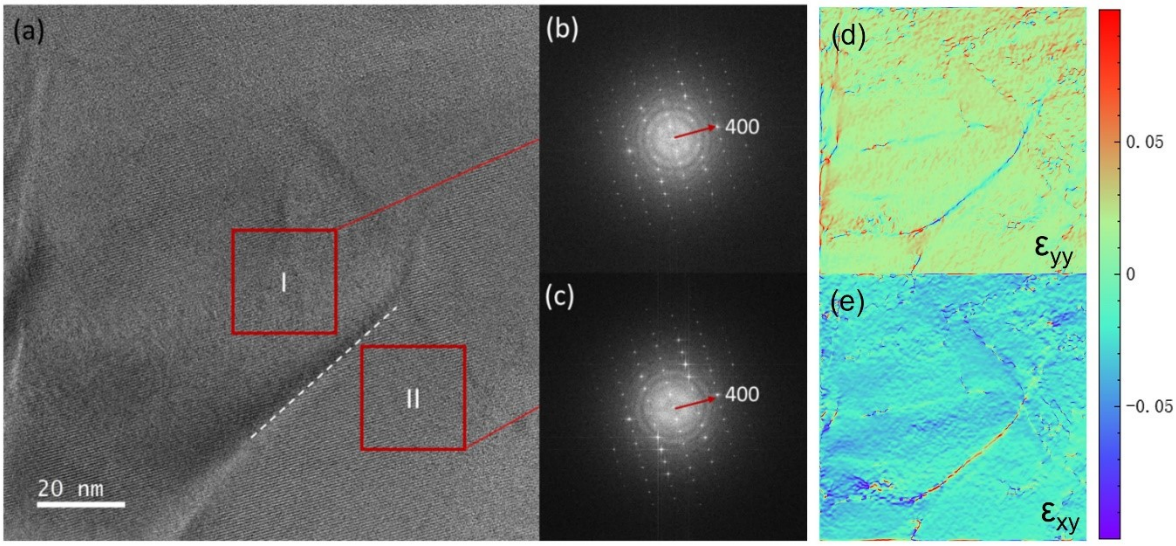

Figure S1. (a) High-resolution TEM image of a wrinkle defect in WTe_2. The image exhibits bending contrast and local orientational deviation of the sample. Region **I** of (a) is oriented close to the [001] zone axis as evidenced by the fast Fourier transform (FFT) pattern shown in (b). The local orientation in region **II** in (a) is bent away from the [001] zone axis, as evidenced by the loss of mirror symmetry of the intensity distribution along the [400] direction in the FFT pattern in (c). The local bending of the sample induces a local strain field in the sample as shown in (d) and (e). The strain field of the sample is calculated from the HRTEM image using the Strain++ software[1] with a geometric phase analysis algorithm.

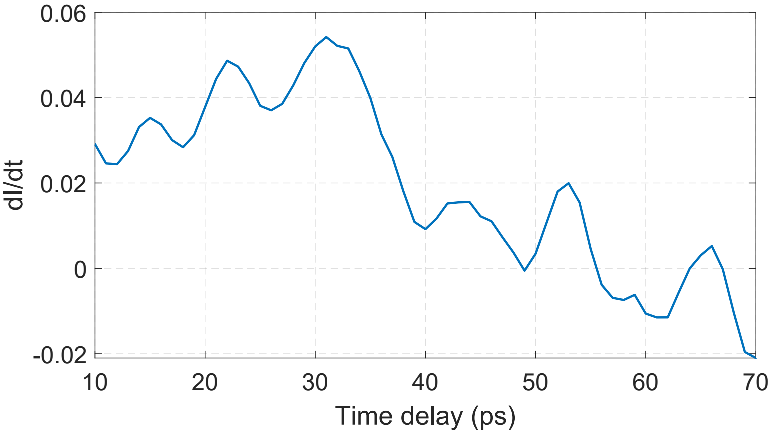

Figure S2. First order derivative of the 120 intensities in Figure 1(f) (see main text) at 2.5 mJ/cm^2 pump fluence. The curve was smoothed by a Gaussian-weighted moving average filter (10 ps filter window). From the first derivative we can identify a decrease in the rate of intensity increase after 30 ps (see discussion around Figure 1(f) in the main text) and that the 120 intensity begin to decrease at around 60 ps.

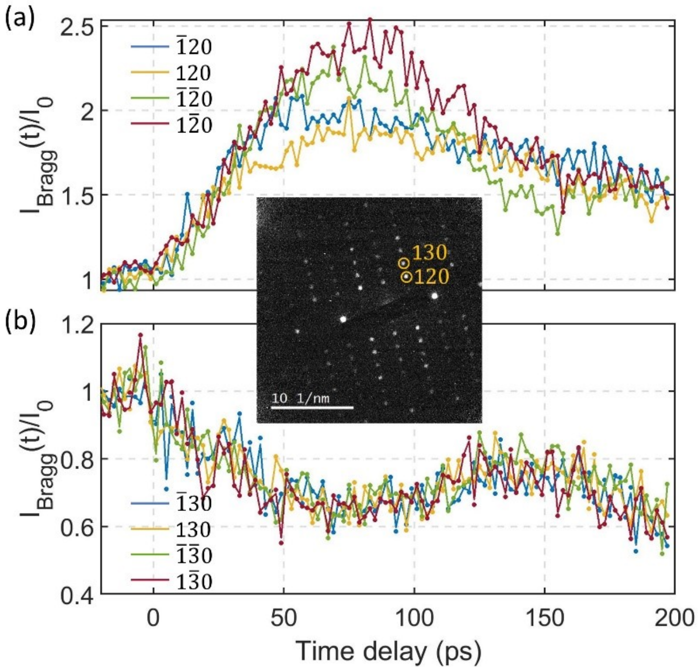

Figure S3. Temporal evolution of the intensity of the (a) 120 and its symmetric diffraction spots and (b) 130 and its symmetric diffraction spots. The pump laser fluence was 1.3 mJ/cm^2. The inset is a diffraction pattern obtained at the [001] zone axis. The 120 and 130 diffraction spots are indicated. From the curves in (a) and (b), we observe that the symmetric diffraction spots exhibit the same evolution profile as a function of time.

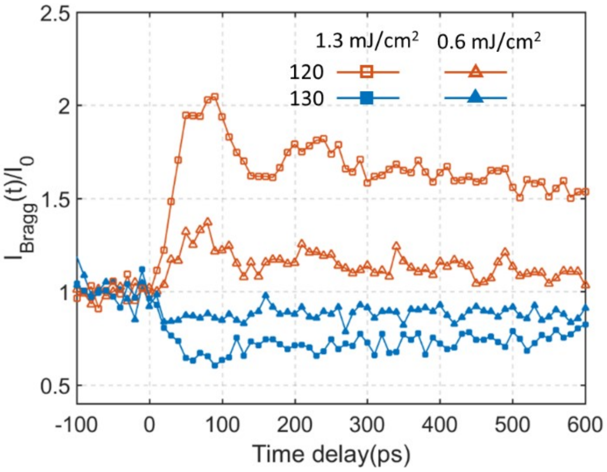

Figure S4. Time resolved intensity of the 120 and 130 spots in a sample with wrinkle defects at an extended time scale for pump fluences of 1.3 mJ/cm^2 and 0.6 mJ/cm^2. As a supplementary of Figure 1(f) in main text, includes results up to 600 ps delay time. On this time scale the decrease in intensity after 80 ps, following a period of intensity oscillations, in the 120 diffraction spots is observed.

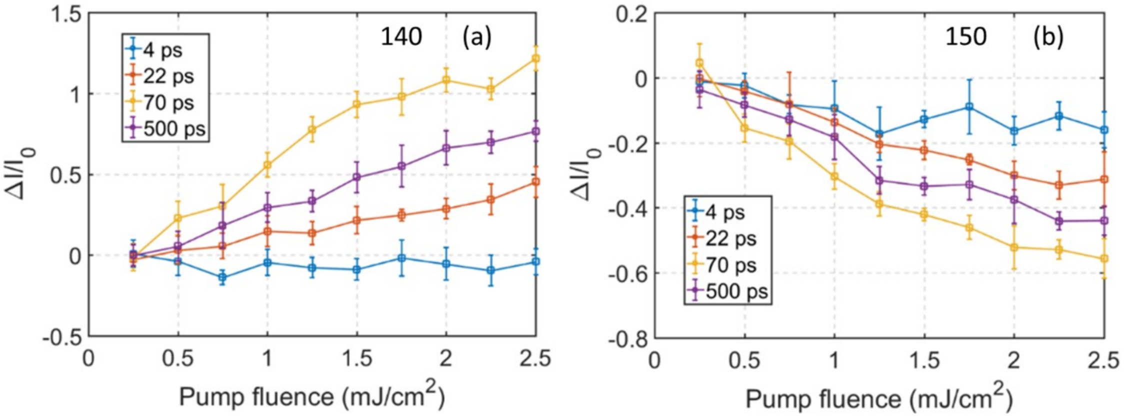

Figure S5. Pump fluence dependent intensity change of (a) the 140 spot and (b) the 150 spot from a sample containing wrinkle defects. The intensity change of the 140 spot as a function of pump fluence is similar to what is observed for the 120 spots. The 150 spot shows similar behavior to that of the 130 spots. The opposite dependence of 140 and 150 spots as a function of pump fluence indicates a photo-induced structure change in *Td*-WTe_2 at 70 ps. Between 70 ps and 500 ps, the intensity increase of the 140 spot and the decrease of the 150 spot reveals a recovery of the structure. Data points are averaged from five experimental data sets and the error bars represent the standard deviation.

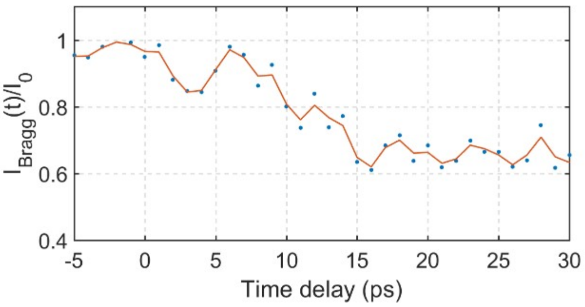

Figure S6. Diffraction intensity evolution of the 130 spot from a sample with wrinkle defects at 2.5 mJ/cm^2 pump fluence. Within this time range the sample reaches a quasi-equilibrium state at ~15 ps. This quasi-equilibrium structure was identified as the 1*T** phase in a recent study [1].

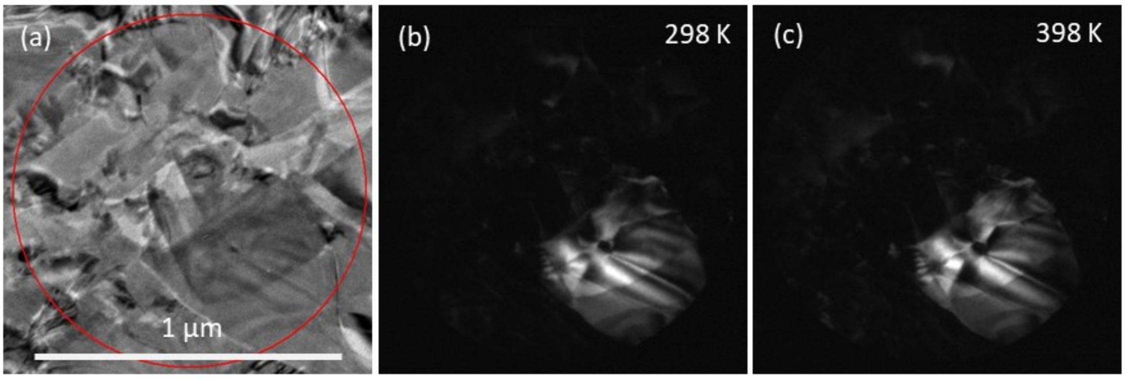

Figure S7. (a) Bright field image a WTe_2 sample with wrinkle defects. (b) Dark field image from selecting the 120 diffraction spot collected at 298 K. (c) Dark field image from the same area as in (b) collected at 398 K. A comparison of the dark field images (b) and (c) shows no significant change. This implies that in the photo-driven experiments observed structural transition is not thermally activated at 398 K (a temperature equivalent to the temperature after time zero in photo-driven experiments). The change in microstructure observed in the photo-driven experiments must therefore be explained as a non-equilibrium structure modulation.

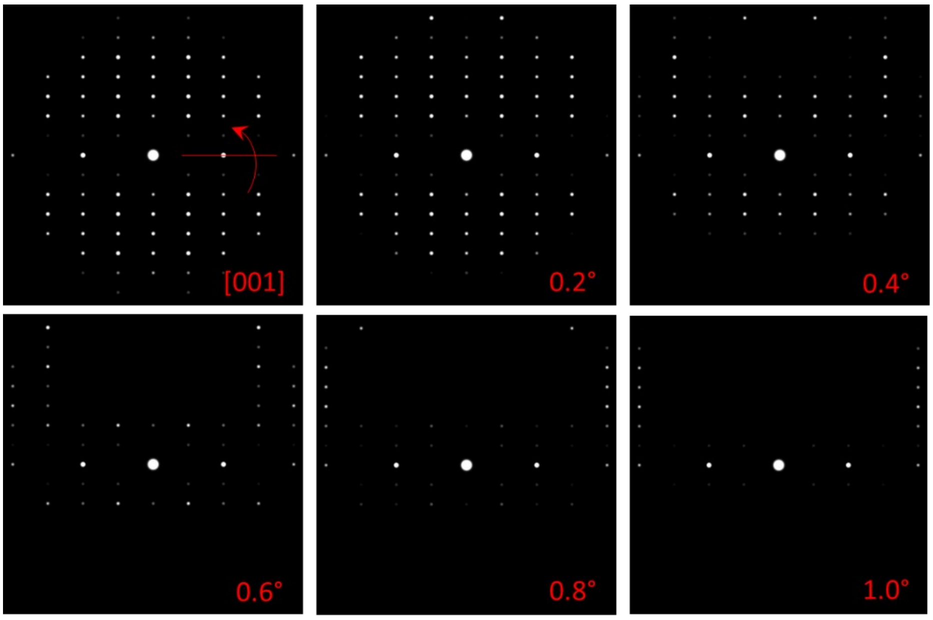

Figure S8. Simulated *Td*-WTe_2 diffraction patterns at the [001] zone axis and at indicated angles off the [001] zone axis, by tilting the sample along the **a** axis. The simulations show that the observed time resolved changes in diffraction intensity after photo-excitation cannot be explained by sample tilt but must be explained by a change in structure. In the simulated diffraction patterns, we observe a decrease in the intensities of the 120, 130, 140 and 150 spots with increasing of tilting angles. This do not match the photo-induced results where the 120 and 140 spots show a reverse trend with that of the 130 and 150 diffraction spots. Based on this, it can be concluded that the photo-induced structural dynamics in the sample with wrinkle defects must include a change in structure.

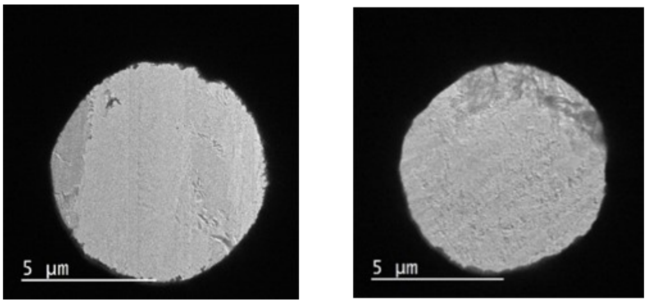

Figure S9. Low magnification TEM micrographs of regions with low (left) and high (right) defect density. The borders in the images are the circular openings of the copper grid.

Reference

1. Hÿtch, M. J., Snoeck, E., & Kilaas, R., Quantitative measurement of displacement and strain fields from HREM micrographs. *Ultramicroscopy* **1998**, *74*(3), 131-146.

2.Ji, S.; Grånäs, O.; Weissenrieder, J., Manipulation of Stacking Order in *Td*-WTe_2 by Ultrafast Optical Excitation. *ACS Nano* **2021,** *15* (5), 8826-8835.
